# Factors controlling the spatial distribution of soil organic carbon in Daxing’anling Mountain

**DOI:** 10.1038/s41598-020-69590-y

**Published:** 2020-07-29

**Authors:** Junyao Li, Dongyou Zhang, Mei Liu

**Affiliations:** Heilongjiang Province Key Laboratory of Geographical Environment Monitoring and Spatial Information Service in Cold Regions, Harbin Normal University, Harbin, 150025 People’s Republic of China

**Keywords:** Ecology, Environmental sciences

## Abstract

Daxing’anling Mountain, in the northeastern part of China, contains a large amount of soil organic carbon (SOC). Using data including topography, climate, and vegetation, the spatial distribution of SOC content was modeled using classical and geography-based statistics, as well as a geographically weighted kriging model. The study findings include: (1) SOC content generally ranges 40–70 g/kg, with high SOC content in the southwest and low SOC content in the southeast; (2) Results of principal component analysis suggested the normalized difference vegetation index is the best predictor of patterns in SOC; and (3) The geo-weighted regression Kriging model well reflects factors influencing spatial distribution of SOC content. This study provides important baseline information for environmental protection in the Daxing’anling Mountain area, as well as general information as to important factors that mediate this important reservoir of soil carbon.

## Introduction

As the largest terrestrial ecosystem carbon pool, soil organic carbon (SOC) plays a critical role in the Earth’s climate. Soil carbon storage is 2–3 times that of the global terrestrial vegetation carbon pool^1–3^. Current studies have shown that SOC is an atmospheric CO_2_ sink and SOC pools can help mediate atmospheric CO_2_ concentrations and mitigate global warming^4^. Frozen soil refers to any rock and soil below 0 ℃ that contains ice. Generally, it can be divided into short-term frozen soil (hours or days to two weeks), seasonally frozen soil (2 weeks to several months) and permafrost (refers to a layer of frozen and unmelted soil that lasts 2 years or more). Studies have shown that half of the global SOC is in frozen soil^5^ and a large amount of soil is stored in permafrost regions. Climate warming and degradation of permafrost cause the long-term storage of SOC to be released, changing the carbon cycle of the original permafrost area and perhaps accelerating climate warming^6^.

For the Qinghai-Tibet Plateau, with the largest area of frozen soil in China’s low latitudes, the thickness of the permafrost active layer is increasing while the area of frozen soil is decreasing^7^. Research by Plaza et al.^8^ found that with the degradation of permafrost, the rate of organic carbon loss was as high as 4.5% a^−1^. Daxing’anling Mountain is located in northeastern China, on the southern edge of the high-latitude permafrost region of Eurasia. Frozen soil is mainly permafrost at high latitudes. It contains a key state-owned natural forests area and contains a large amount of soil organic carbon.

Regionally, SOC is critical for agriculture and environmental ecology^9^, and its content directly affects the function and sustainable utilization of soil ecosystems^10^. The spatial distribution characteristics of SOC content are affected by many environmental cofactors and their variability has different characteristics at different scales^11,12^. In recent years, the Daxing’anling Mountain range has experienced severe degradation in ecological function^13^. In this context, we studied factors influencing the spatial distribution of SOC and the main factors controlling it in the Daxing’anling Mountain range.

## Study area

The study area was the Daxing’anling Mountain range in the northeast region of China. The geographic coordinates are 121°12′–127°00′ E, 50°10′–53°33′ N^14^ and the total area is 8.35 million km^2^^15^,the east–west length is greater than the north–south expanse. It is mainly composed of middle-lower mountains and tundra that are higher in the northeast and lower in the northwest. The average altitude of the area is 573 m and the highest altitude 1528 m. The altitude of the western and central parts of Huzhong District, the Xinlin District, and Tahe County is 300–500m^16^. The average slope is 12° ^15^.

In the study area, water resources are rich, with nearly 150 rivers, including the Huma, Pangu, Naduli, Xiergen, Gan, Emuer, and Duobukuer, among others. Vegetation diversity is low, mainly composed of the main foundation species *Larix gmelinii* Kuzen in the northern mountains, accounting for 75% of total cover^17^. Other tree species include Pinus *sylvestris* var. *mongolica* Litv., *Pinus pumila*, *Betula platyphylla* Suk, and *Picea asperata* Mast.

**Figure 1:**
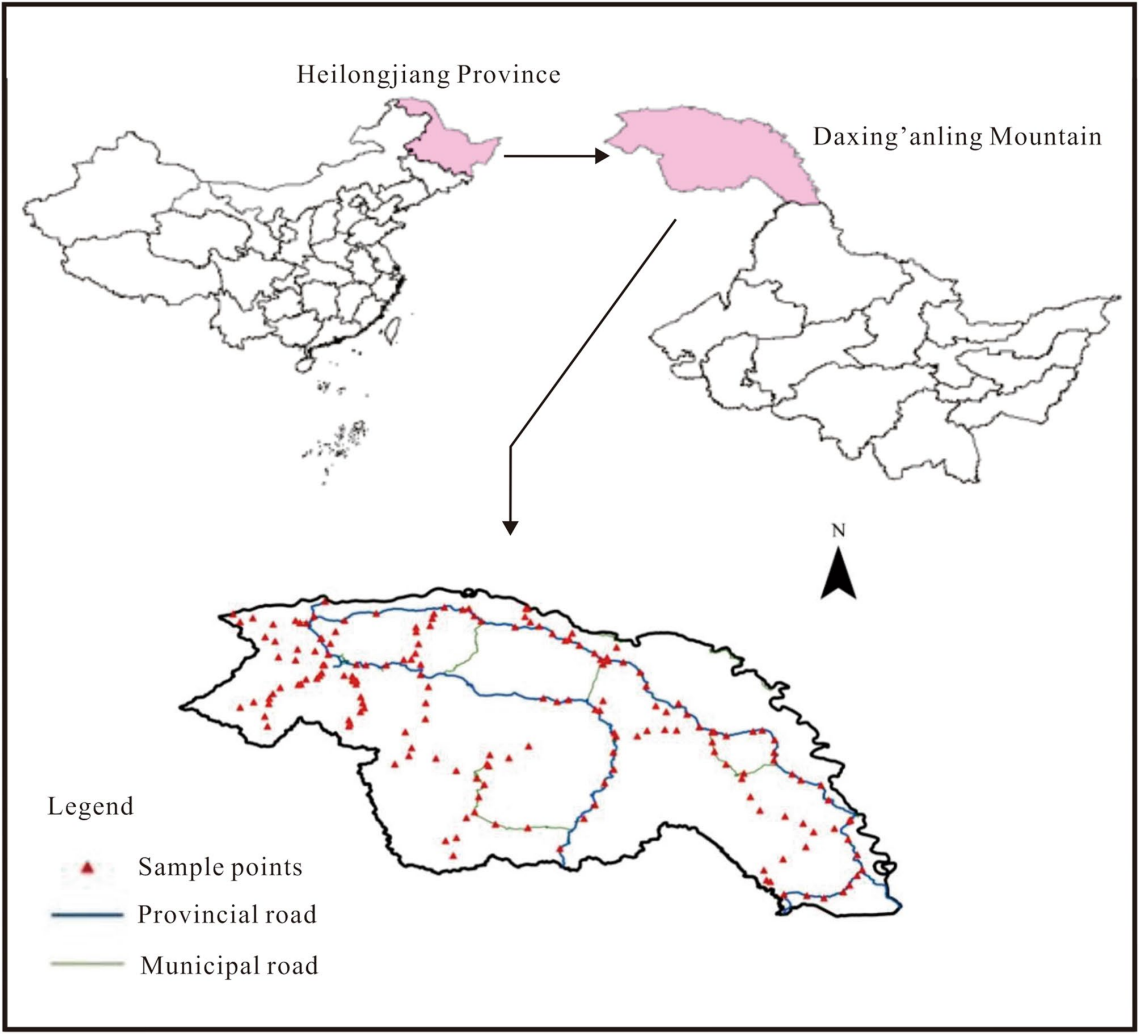
Distribution of sampling locations across the Daxing’anling Mountain in northeastern China. The map was generated by software ArcGIS 10.1 (https://www.esri.com/) by Junyao Li & Mei Liu.

Experimental samples were collected in July 2018. Sampling points were arranged according to the land use map of the frozen soil area of Daxing’anling Mountain. A total of 180 sample points was collected using a soil drill and other tools and we ensured that sites were distributed across the study area (Fig. 1).

## Results

### Spatial distribution of SOC content

**Figure 2:**
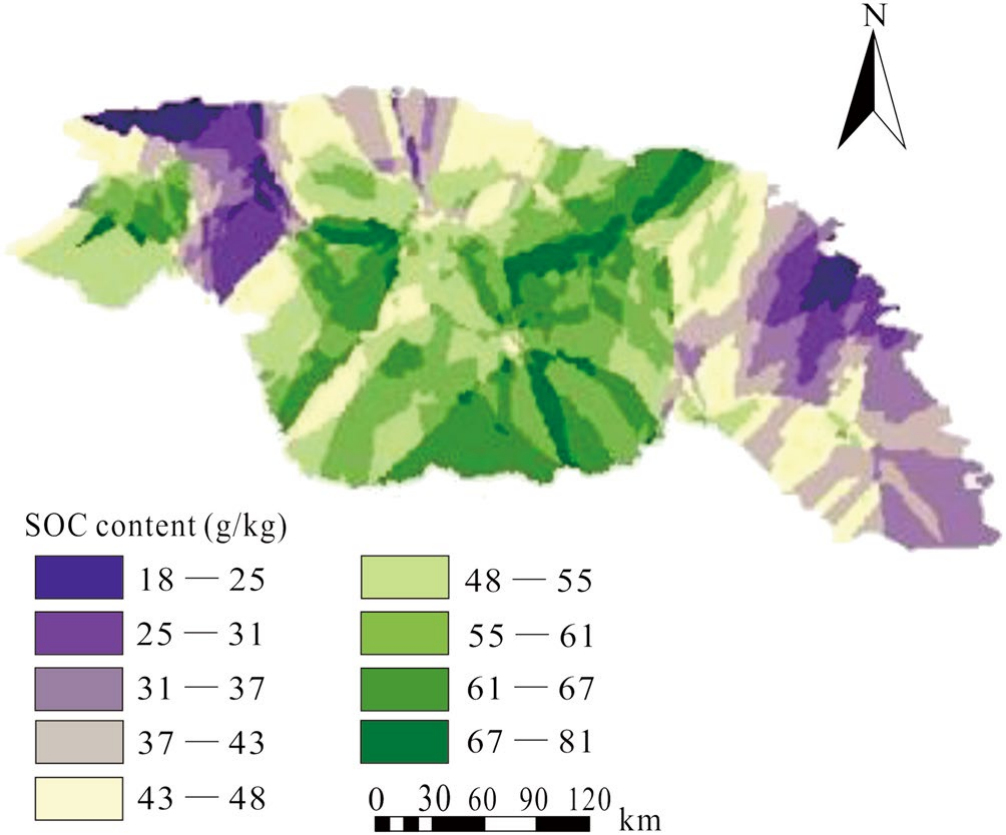
Spatial distribution of SOC content in the Daxing’anling Mountain range. Select the ordinary Kriging model and perform Kriging interpolation on the sampling point data to obtain the spatial distribution of SOC content. The figure was generated by ArcGIS 10.1.

Interpolation parameters were obtained based on a geostatistical semi-variance function method, and results of the parameters obtained by Kriging to get a better overall model. The SOC content in Daxing’anling Mountain is transformed from discrete point information to continuous surface information, and the spatial distribution characteristics of SOC content could then be further analyzed. Through this approach, we can use fewer sampling points to predict spatial information of soil properties in the entire Daxing’anling Mountain area, as shown in Fig. 2. Results suggest that prediction accuracy is high. It can be seen in the map of the spatial distribution that SOC content is heterogeneous, lower in the northwest and southeast. SOC content generally ranges from ~ 40–70 g/kg.

### Principal component analysis of SOC and auxiliary environmental variables

To determine the contributions of environmental auxiliary variables to SOC, correlations between SOC and environmental auxiliary variables were analyzed. Auxiliary environmental variables, their abbreviations and results are displayed in Table 1, showing a range of positive and negative correlation coefficients.

**Table 1:** SOC content correlation with environmental variables in the Daxing’anling Mountain range.

Environmental auxiliary variables	Abbreviation	SOC content correlation coefficient
Normalized Difference Vegetation Index	NDVI	0.54
Integrated land use index	La	0.51
Slope	S	0.44
Aspect	A	0.41
Elevation	H	0.42
Profile curvature	Cp	0.23
Plan curvature	Ct	− 0.16
Topographic Wetness Index	TWI	0.34
Convergence of confluence	Cc	0.34
Surface temperature	St	0.02

The SOC content in Daxing’an Mountain is taken as the dependent variable, and ten influential factors such as quantitative normalized difference vegetation index, integrated land use index, slope, aspect, elevation, profile curvature, plan curvature, topographic wetness index, convergence of confluence, and surface temperature are taken as independent variable, using X1 X2……X10 named. Based on ten independent variables and principal component analysis, the eigenvalues, contribution rates and cumulative contribution rates of the ten environmental auxiliary factors in this paper are obtained, and the main influencing factors of SOC content are analyzed and determined. The results are shown in Table 2.

**Table 2:** Influence factor eigenvalue and principal component contribution rate.

Impact factor	Component	Eigenvalue	Contribution rate (%)	Cumulative contribution rate (%)
NDVI	1	2.0	20.4	20.4
La	2	1.8	18.5	38.9
S	3	1.4	14.2	53.1
A	4	1.0	10.2	63.3
H	5	1.0	10.2	73.5
Cp	6	0.9	8.8	82.2
Ct	7	0.7	7.1	89.3
TWI	8	0.6	6.3	95.6
Cc	9	0.3	2.7	98.3
St	10	0.2	1.7	100.0

The cumulative contribution of the first, second, third, fourth, and fifth principal components is 73.5%. The top five principal components met the requirements of the Kaiser criterion, which suggests strong explanatory power for the SOC variation for Daxing’anling Mountain.

The first principal component is NDVI, whose contribution rate is 20.4%. The second principal component is the land use comprehensive index (18.5%), indicating that the change of soil organic carbon content in Daxing’anling Mountain is related to residential land, roads, rivers, and green space. The third principal component is the slope (14.2%), the fourth principal component is the aspect (10.2%), and the fifth is the elevation (10.2%). Indicating that the topographic changes in Daxing’an Mountain range are correlated with the SOC content and will have a certain influence on it.

### Evaluation of the geographically weighted regression Kriging model

Using geographically weighted regression (GWR) and multiple linear regression (MLR) models for analysis, the same auxiliary variables were selected to compare the two models. Bandwidth was set according to the modified Akaike-information criterion^18^ as shown in Table 3. The R^2^ value of the GWR model (0.47) is higher than that of the MLR model (0.30), which suggests the GWR model is better in identifying factors influencing SOC spatial distribution. Furthermore, the AICC value of the GWR model is lower than that of the MLR model, suggesting a better model fit^18^.

**Table 3:** Diagnostic information of the MLR and GWR residual models for SOC.

**Model**	**AICC**	**R**^**2**^	${\mathbf{R}}_{{\varvec{a}}{\varvec{d}}{\varvec{j}}}^{2}$
Regression model	1786.9	0.4	0.1
Geographic-weighted regression	1784.9	0.5	0.3

Five-fold cross-validation was used to verify and evaluate the interpolation accuracy of the geographically weighted regression kriging model (GWRK) and the regression kriging model (RK). Soil sample data were divided randomly into five parts, and then one part was designated as a verification set and was only used for evaluation of model accuracy. The remaining ones were used for spatial interpolation in model formation. The above process was carried out five times to obtain the simulated value of SOC of the data set. The average error and correlation coefficients are used to evaluate and verify the prediction accuracy of each model. Results show that the RMSE value of the GWRK model (3.5) is less than that of the RK model (3.8), suggesting the GWRK model is superior. This also suggests there are many factors to consider when studying the auxiliary variables of spatial distribution characteristics of SOC content, which requires us to consider not only the fitting of environmental auxiliary variables but also additional spatial and structural information.

### Factors controlling SOC content spatial distribution

**Figure 3:**
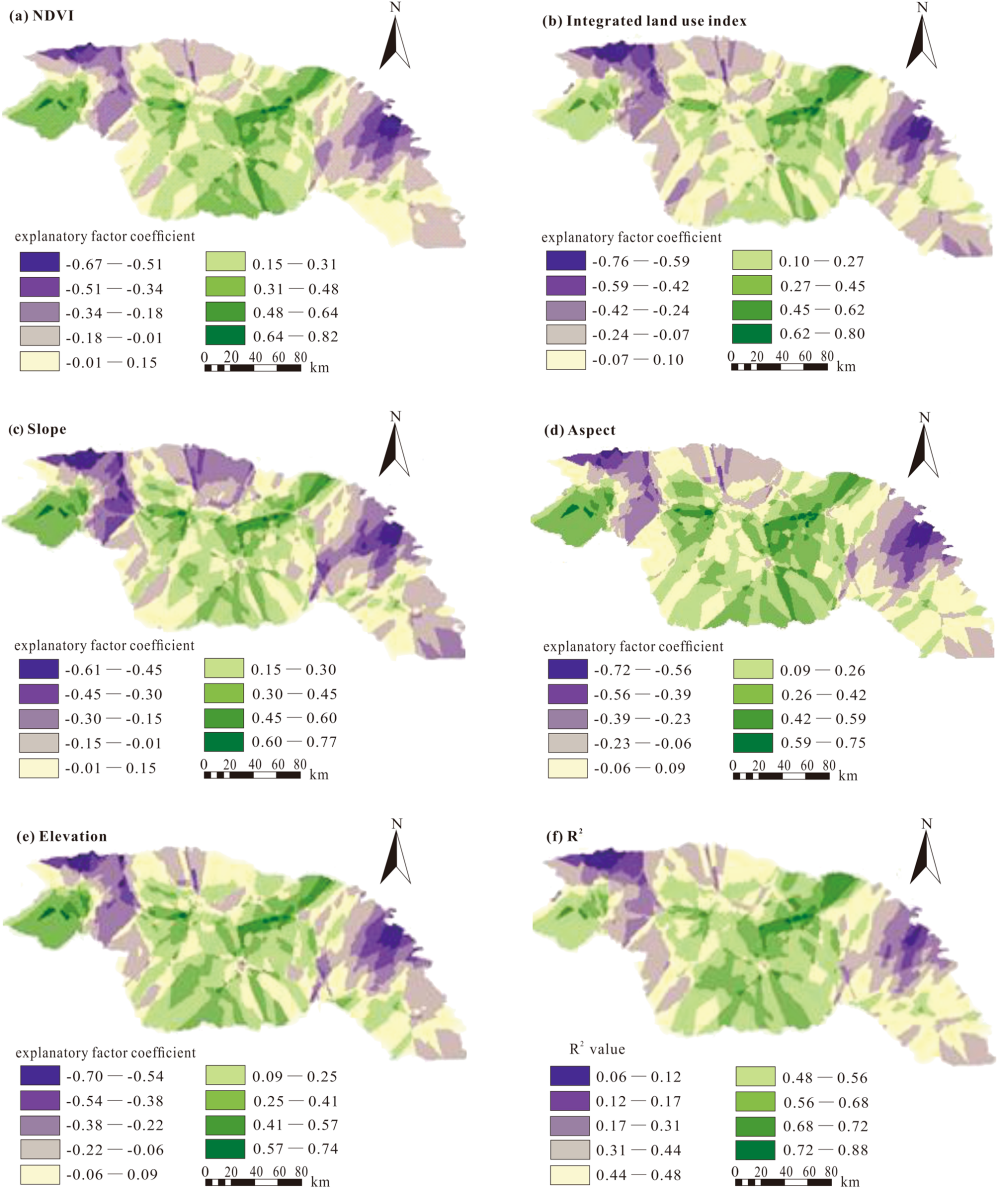
Explanatory variable coefficients in the GWRK model for SOC and spatial distribution of R^2^. Use the GWRK model to analyze the influencing factors of SOC and obtain the fitting result graph of the GWRK model. (**a**) NDVI, (**b**) Integrated land use index, (**c**) Slope, (**d**) Aspect, (**e**) Elevation, (**f**) R^2^. All figures were generated by ArcGIS 10.1.

The spatial variation of SOC content, which is related to the environmental auxiliary variables, has predictable geospatial characteristics. Five key indicators (those that loaded high on the first five PCA axes) were identified: normalized vegetation difference index, integrated land use index, slope, aspect, and elevation. These five factors and results of GWRK model fitting were used to estimate the spatial distribution of SOC content and results are shown in Fig. 3. Coefficients of explanatory factors vary with location.

The coefficient with the largest absolute value is the main controlling variable in a geographical location^19^. Compared with the other four environmental explanatory factors, absolute values of NDVI coefficients are highest. The influence of NDVI on the spatial distribution of SOC content decreased from the mideast to the northwest and the southeast. This suggests that the higher the vegetation coverage, the greater the control on the SOC content. The other four environmental auxiliary factors play a more secondary role.

The integrated land use index ranks second in importance to NDVI. Its influence on SOC spatial distribution is reflected in the northeast, northwest, and southeast. In the northeast part of the study area, La is positively correlated with SOC content which suggests vegetation cover will promote the accumulation of SOC. In the northwest and southeast of the study area, the integrated land use index (La) is negatively correlated with SOC.

The slope and aspect have a major influence on the spatial distribution of SOC content in the central and western areas. Some low-slope areas are disturbed by human activities. When the slope increases limiting human activities, the impact of slope on SOC is positively correlated. The sunny slope side is conducive to SOC accumulation. In the western and central areas, the elevation is positively correlated with SOC content. As the altitude increases, the vegetation coverage is higher which will promote the accumulation of SOC. In the eastern areas, the elevation is negatively correlated with SOC because of farming and other factors.

Regions with the best model fits are distributed in the eastern and central parts of the study area, whereas regions with weaker fits are in the northwest.

## Discussion

The response of permafrost organic carbon to climate warming is a matter of general concern as it will lead to environmental changes affecting production, environment, and socioeconomic security^20,21^. Some studies have found that the physical and chemical properties of soil and the distribution of surface vegetation are the most direct driving factors affecting the spatial variability of soil organic carbon^22^. In McGrath et al.^23^ research on organic carbon in grassland soils in Ireland, it was found that rainfall is a key factor affecting its spatial distribution. Li^24^ found that the average annual temperature and rainfall both had a significant impact on the organic carbon content of farmland soil in China. Huang's^25^ found that soil bulk density and topographic altitude mainly affected SOC content, while clay content and annual average temperature had little effect. Chen et al.^26^ research on soil organic carbon in natural ecosystems in northern China found that higher vegetation coverage is beneficial to soil organic carbon accumulation. In this study, results of GWRK fitting shows that the absolute value of NDVI factor coefficient is the highest. The NDVI index reflects vegetation coverage, biomass, and vegetation growth status^27^. The index shows a positive correlation between vegetation and soil organic carbon, likely because of the accumulation of surface soil litter^28^.

In the SOC analysis at small and medium scales, scholars often focus on the linear relationship between influencing factors and soil organic carbon, not incorporating spatial differences. Conventional linear regression models may mask the true characteristics of spatial data^29^. The geographically weighted regression model (GWR) is a supplement and extension to the general linear model and has a wide range of applications in environmental fields and soil analysis^19,30,31^. There is a difference between the predicted value calculated by the two models and actual values, i.e., the residual error. Some researchers use residual error information of the models for spatial prediction and combine the results of the two methods for improved prediction capability^32^. In Sun's research on forest carbon storage in Maoershan, the prediction accuracy of the GWRK model is higher^33^. In this study, the results of the two models minimize local variability and residual effects in the study area. The GWRK model was applied to account up for deficiencies of MLR and GWR, and the SOC content prediction was more accurate as a result.

## Methods

### Soil sampling and laboratory analysis

Soil sampling depth was 0–20 cm, and one sample was obtained by five-point sampling within a 15 × 15 m area. The five-point sampling method refers to first determining the center point of the diagonal as the center sampling point, and then selecting four points on the diagonal line that are equal in distance from the center sample. Soil samples were placed in a cloth bag and labeled, and the temperature, longitude, latitude, and elevation data of soil samples were recorded. Soil samples were air-dried, ground, and sifted as a pretreatment. They then were weighed, 0.1 mol/L hydrochloric acid was added to remove inorganic carbon, and samples re-dried. SOC was determined by a German Jena multi N/C 3100 TOC analyzer.

### Additional environmental data

Terrain, climate, vegetation, and land use were selected as environmental auxiliary data to examine spatial variation of SOC in the Daxing’anling Mountain range. Generating derivatives is commonly used in the topographic analysis, and the factors describing these features are called topographic factors^34^ Digital elevation models (DEM) use terrain elevation data to create a digital simulation of the terrain surface^35^. Topographic data used were compiled from USGS and auxiliary data, such as slope and aspect, and were extracted using ArcGIS software.

In the analysis of land use and SOC, quantitative data are critical. To this end, we employed the comprehensive index of land use proposed by Zhuang et al.^36^.

The normalized difference vegetation index (NDVI) represents plant growth form and the spatial distribution density of vegetation. The formula for obtaining NDVI is:

1 $${\text{NDVI}} = ({\text{NIR}} - {\text{RED)/(NIR}} + {\text{RED)}}$$

where NIR is the near-infrared band and RED is the infrared band.

Image data were obtained from Landsat8 in July 2018, and NDVI and land use type were processed using these. The environmental auxiliary data are shown in Table 4.

**Table 4:** Variables used for quantitative models of SOC in the Daxing’anling Mountain range. DEM refers to digital elevation models. OLI (Operational Land Imager) is a land imager in Landsat 8.

No.	Variables	Abbreviation	Source
1	Slope	S	DEM
2	Aspect	A	DEM
3	Elevation	H	This study
4	Plan curvature	Ct	DEM
5	Profile curvature	Cp	DEM
6	Surface temperature	St	This study
7	Convergence of confluence	Cc	DEM
8	Topographic Wetness Index	TWI	DEM
9	Integrated land use index	La	LAND SAT 8 OLI
10	Normalized Difference Vegetation Index	NDVI	LAND SAT 8 OLI

### The Kriging interpolation method

To obtain an intuitive SOC spatial distribution, the ordinary Kriging interpolation method was used. One advantage of this method is the inclusion of adjacent sample information. By using structural characteristics of the original data, a linear, unbiased, optimal estimation of values for sites not sampled in the study area can be established. The formula is:

2 $${\text{Y}}\left( {{\text{X}}_{0} } \right) = \mathop \sum \limits_{{{\text{i}} = 1}}^{{\text{N}}} {\uplambda }_{{\text{i}}} \cdot {\text{Y}}\left( {{\text{X}}_{{\text{i}}} } \right)$$

where Y(X_0_) represents the value of the unsampled point, ${\uplambda }_{\mathrm{i}}$ is the weight of the sampled point relative to the unsampled point, and Y(X_i_) is the value of the known sample point adjacent to the sampled point.

### Principal component analysis

Since there are multiple variables in this study, a principal component analysis was applied. Due to the high correlation among variables, it is necessary to simplify into fewer predictive axes. To achieve data reduction, principal components were extracted representing the original variables (with a different relative importance of each variable, or their Eigenvalues) while ensuring that original information is best conserved.

### Compound model construction for spatial prediction of SOC content

Multiple linear regression models (MLR) and geographically weighted regression models (GWR) can be used to predict spatial variation, distribution trends, and driving factors of SOC content. In our study, uncertainties in the simulation of spatial distribution trends, the apparent randomness of influencing factors, the geographical location of the samples, their spatial structure, local site distribution characteristics, and key characteristics of residuals are considered. To this end, the regression Kriging (RK) model and GWR extension model were utilized, which combined the results of the MLR models with the regression-residual interpolation hybrid-space modeling method, i.e., a geographically weighted regression kriging model (GWRK) based on GWR interpolation. These models provided a comprehensive approach to reflect the spatial distribution characteristics of SOC in Daxing’anling Mountain.

## References

[1] Larionova AA (2015). Assessing the stability of soil organic matter by fractionation and C-13 isotope techniques. Eurasian Soil Sci..

[2] Schlesinger WH (1997). Biogeochemistry: an analysis of global change. Bull. Am. Meteor. Soc..

[3] Lal R (2004). Soil carbon sequestration impacts on global climate change and food security. Science.

[4] Schmidt MW (2011). Persistence of soil organic matter as an ecosystem property. Nature.

[5] Carvalhais N (2014). Global covariation of carbon turnover times with climate in terrestrial ecosystems. Nature.

[6] Ma Q, Jin HJ (2020). Impacts of climate warming on soil organic pools in permafrost regions. J. Glaciol. Geocryol..

[7] Wu Q, Zhang T (2010). Changes in active layer thickness over the Qinghai-Tibetan Plateau from 1995 to 2007. J. Geophys. Res. Atmos..

[8] Plaza C (2019). Direct observation of permafrost degradation and rapid soil carbon loss in tundra. Nat. Geosci..

[9] Zhou, Y. High-resolution mapping of soil organic carbon and its spatial multiscale controlling factors in Tibet. Ph.D., Zhejiang University, Hang Zhou (2018)

[10] Zou, R. Y. Spatial variation of soil organic carbon and it’s influence factors of cropland around Poyang Lake. Master's Degree, Jiangxi University of Finance and Economics, Nan Chang (2019).

[11] O’rourke SM, Angers A, Holden NM, McBratney AB (2015). Soil organic carbon across scales. Glob. Change Biol..

[12] Zhou Y, Biswas A, Ma ZQ, Lu YL, Chen QX, Shi Z (2016). Revealing the scale-specific controls of soil organic matter at large scale in Northeast and North China Plain. Geoderma.

[13] Wu, Y. X. Ecological research governance issues to enhance the Daxing’anling Mountain. Master's Degree, Harbin University of Commerce, Harbin (2016).

[14] Yang, G. The impact of climate change on forest fire in China’s boreal forest. Ph.D., Northeast Forestry University, Harbin (2010).

[15] Liu, K. Z. Fire environment and forecasting models of summer forest fire in Daxing’anling Mountain district. Ph.D., Chinese Academy of Forestry, Peking (2018).

[16] Zhang, L. B. Study on spatial variability of forest health in the Daxing’anling Mountains of Inner Mongolia. Master’s Degree, Inner Mongolia Agricultural University, Inner Mongolia (2018).

[17] Yu, Y. Analysis of impact factors on larch caterpillar’s occurrence in the Great Xing’an Mountains. Master’s Degree, Shenyang Normal University, Shen Yang (2016).

[18] McQuarrie ADR, Tsai CL (1998). Regression and time series model selection. World Sci..

[19] Yan, A. Spatial distribution and storages estimation of soil organic carbon and soil inorganic carbon in Xinjiang, China. Ph.D., China Agricultural University, Peking (2015).

[20] Schuur EA (2015). Climate change and the permafrost carbon feedback. Nature.

[21] Yumashev D (2019). Climate policy implications of nonlinear decline of Arctic land permafrost and other cryosphere elements. Nat. Commun..

[22] Ruiz-Colmenero M, Bienes R, Eldridge DJ, Marques MJ (2013). Vegetation cover reduces erosion and enhances soil organic carbon in a vineyard in central Spain. CATENA.

[23] McGrath D, Zhuang GS (2003). Spatial distribution of soil organic carbon concentrations in grassland of Ireland. Appl. Geochem..

[24] Li JQ, Li ZL, Jiang GF, Cheng H, Fang CM (2016). A study in soil organic in plough layer of China's arable land. J. Fudan Univ. (Nat. Sci).

[25] Huang YM, Lee XQ, Yang F, Huang DK, Xing Y (2016). Spatial variation of soil organic in karst forests of the southwestern China and its affecting factors. Earth Environ..

[26] Chen XT, Xu TL, Li XJ, Zhao AH, Feng HY, Chen BD (2019). Soil organic carbon concentrations and the influencing factors in natural ecosystems of northern China. Chin. J. Ecol..

[27] Prince SD (1991). A model of regional primary production for use with coarse resolution satellite data. Int. J. Remote Sens..

[28] Chaplot V, Bouahom B, Valentin C (2010). Soil organic carbon stocks in Laos: spatial variations and controlling factors. Glob. Change Biol..

[29] Qin WZ, Wang JM, Liu ML (2005). Spatial nonstationarity of geographically weighted regression analysis of spatial date. J. Liaoning Normal Univ. (Nat. Sci. Ed.).

[30] Li L, Yao YF, Qin FC, Zhang ML, Gao YH, Chang WD (2016). Analysis on influence factors of soil organic carbon density using a geographically weighted regression model. Sci. Technol. Rev..

[31] Yang SH (2015). The spatial variability of soil organic carbon in plain-hills transition belt and its environmental impact. China Environ. Sci..

[32] Zeng CY (2016). Mapping soil organic matter concentration at different scales using a mixed geographically weighted regression method. Geoderma.

[33] Sun, Y. S. Spatial distribution of forest carbon storage in Maoershan region based on GWRK model. Master's Degree, Northeast Forestry University, Harbin (2019). 10.13287/j.1001-9332.201905.024 31107021

[34] Cui, T. G. Analysis of terrain factors in sugarcane area based on DEM—Take Liujiang county as an example. Master's Degree, Guangxi University, Nan Ning (2018).

[35] Tang, G. A., Liu, X. J. & Lv, G. N. Principles and methods of digital elevation model and geoscience analysis. 1–33 (Science Press, Peking, China, 2005).

[36] Zhuang DF, Liu JY (1997). Study on the Model of regional differentiation of land use degree in China. J. Nat. Resour..

